# Functional Roles of Bromodomain Proteins in Cancer

**DOI:** 10.3390/cancers13143606

**Published:** 2021-07-19

**Authors:** Samuel P. Boyson, Cong Gao, Kathleen Quinn, Joseph Boyd, Hana Paculova, Seth Frietze, Karen C. Glass

**Affiliations:** 1 Department of Pharmaceutical Sciences, Albany College of Pharmacy and Health Sciences, Colchester, VT 05446, USA; sam.boyson@acphs.edu; 2 Department of Pharmacology, Larner College of Medicine, University of Vermont, Burlington, VT 05405, USA; kathleen.quinn@uvm.edu; 3 Department of Biomedical and Health Sciences, University of Vermont, Burlington, VT 05405, USA; cong.gao@med.uvm.edu (C.G.); joseph.boyd@med.uvm.edu (J.B.); hana.paculova@med.uvm.edu (H.P.); 4 University of Vermont Cancer Center, Burlington, VT 05405, USA

**Keywords:** cancer, bromodomain, epigenetic gene regulation, histone post-translational modifications, chromatin reader, protein-protein interaction network

## Abstract

**Simple Summary:**

This review provides an in depth analysis of the role of bromodomain-containing proteins in cancer development. As readers of acetylated lysine on nucleosomal histones, bromodomain proteins are poised to activate gene expression, and often promote cancer progression. We examined changes in gene expression patterns that are observed in bromodomain-containing proteins and associated with specific cancer types. We also mapped the protein–protein interaction network for the human bromodomain-containing proteins, discuss the cellular roles of these epigenetic regulators as part of nine different functional groups, and identify bromodomain-specific mechanisms in cancer development. Lastly, we summarize emerging strategies to target bromodomain proteins in cancer therapy, including those that may be essential for overcoming resistance. Overall, this review provides a timely discussion of the different mechanisms of bromodomain-containing proteins in cancer, and an updated assessment of their utility as a therapeutic target for a variety of cancer subtypes.

**Abstract:**

Histone acetylation is generally associated with an open chromatin configuration that facilitates many cellular processes including gene transcription, DNA repair, and DNA replication. Aberrant levels of histone lysine acetylation are associated with the development of cancer. Bromodomains represent a family of structurally well-characterized effector domains that recognize acetylated lysines in chromatin. As part of their fundamental reader activity, bromodomain-containing proteins play versatile roles in epigenetic regulation, and additional functional modules are often present in the same protein, or through the assembly of larger enzymatic complexes. Dysregulated gene expression, chromosomal translocations, and/or mutations in bromodomain-containing proteins have been correlated with poor patient outcomes in cancer. Thus, bromodomains have emerged as a highly tractable class of epigenetic targets due to their well-defined structural domains, and the increasing ease of designing or screening for molecules that modulate the reading process. Recent developments in pharmacological agents that target specific bromodomains has helped to understand the diverse mechanisms that bromodomains play with their interaction partners in a variety of chromatin processes, and provide the promise of applying bromodomain inhibitors into the clinical field of cancer treatment. In this review, we explore the expression and protein interactome profiles of bromodomain-containing proteins and discuss them in terms of functional groups. Furthermore, we highlight our current understanding of the roles of bromodomain-containing proteins in cancer, as well as emerging strategies to specifically target bromodomains, including combination therapies using bromodomain inhibitors alongside traditional therapeutic approaches designed to re-program tumorigenesis and metastasis.

## 1. Introduction

Cancer heterogeneity presents major challenges for the development of personalized treatments [1,2,3,4]. Current precision therapies used in the treatment of cancer are designed to exploit a variety of different biological entities characteristic to individual cancer types, such as activated protein kinases, estrogen receptor, and defective DNA repair enzymes [4,5,6]. Understanding the mechanistic details of cancer biology is critical for improving diagnostic tools and for developing new therapeutic interventions. A comprehensive understanding of cancer requires interpretation of molecular intricacies at multiple levels such as genomic, epigenomic, transcriptomic, proteomic, and metabolomic data. With the advent of high-throughput technologies, the availability of multi-omics data has revolutionized our understanding of the disease process and has created new avenues for integrated system-level approaches.

Epigenetic mechanisms are increasingly being recognized as central to the development and progression of cancer. Epigenetic changes are defined as heritable non-genetic mechanisms that impact gene expression [7]. These mechanisms include DNA methylation, post-translational histone modifications (PTMs), and non-coding RNAs (i.e., microRNAs and long non-coding RNA s), which play fundamental roles in essentially all nuclear processes involving DNA, including transcription, DNA replication, and DNA repair [8,9,10]. Improved understanding of the epigenetic mechanisms underlying cancer etiology has resulted in the identification of a number of molecular targets and the development of novel therapeutics and prognostic biomarkers. Many epigenetic inhibitors have emerged as attractive anti-cancer agents in pre-clinical studies [11]. In particular, the recent advent of small-molecule inhibitors that target bromodomains has provided critical insight into our understanding of the biological mechanisms of bromodomain proteins in cancer. Research in this area has focused on the development of inhibitors for the bromodomain and extra-terminal motif (BET) bromodomains; however, more recently, inhibitors targeting the non-BET bromodomains have emerged. These inhibitors have provided new insights into the cellular function of non-BET bromodomain proteins, and our increasing knowledge of bromodomain structure and function has shed light on the structural aspects of the selective histone recognition activities of all bromodomain proteins. The rationale design of a second generation of compounds has produced bromodomain inhibitors that selectively target individual bromodomain proteins, including non-BET bromodomain proteins [12]. However, a great deal remains to be understood regarding the role bromodomain proteins play in cancer progression. Understanding their expression and interaction profiles, and their regulatory roles in chromatin modifiers, could provide additional insight into bromodomain-dependent mechanisms in cancer [13]. The role of bromodomain inhibitors across a variety of cancers may yet be important to refine personalized medicine in cancer treatment.

In this review, we systematically evaluate bromodomain-containing proteins as individual entities of a larger family of epigenetic regulators, highlighting recent advances in our understanding of how recognition of acetylated lysine by the bromodomain influences protein function. Our goal is to outline the function of bromodomain proteins in different biological contexts and to provide insights on the functional role of the bromodomain in these processes from the lessons learned by examining the cellular effects of small-molecule bromodomain inhibitors as potential anti-cancer agents.

## 2. Bromodomains Are Histone Lysine Acetylation Reader Domains

Nucleosomes are the fundamental packaging unit of chromatin, progressively folding DNA into higher-order chromatin structures. A single nucleosome core particle is composed of 147 base pairs of DNA wrapped around a histone octamer containing four subunits of histones H2A, H2B, H3, and H4 [14]. The N-terminal tails of the nucleosomal histone proteins protrude from the core particle and form sites for numerous covalent PTMs, including methylation, acetylation, phosphorylation, and ubiquitination. Among the array of different PTMs, the ε-N-acetylation of lysine residues represents one of the most abundant PTMs on both histone and non-histone proteins [15,16]. The levels of histone acetylation are established by enzymes including lysine acetyltransferases (KATs; also known as histone acetyltransferases (HATs) and histone deacetylases (HDACs)). Widely recognized as fundamental epigenetic marks, the acetylation of histones is well-known to control chromatin structure and function, and it plays a central role in the regulation of gene transcription, DNA repair, and chromatin condensation, as these biological mechanisms are ultimately coordinated by the epigenome-wide pattern of lysine acetylation marks [17].

Bromodomains are protein interaction modules that “read” ε-N-lysine acetylation (Kac) marks [18]. There are other known protein domains that recognize and bind to Kac, including the plant homeodomain (PHD finger) and the Yaf9, ENL, AF9, Taf14, and Sas5 (YEATS) domains [19,20]. However, their primary targets encompass additional PTMs including methylated and crotonylated lysine, respectively [21,22]. Through their ability to read a variety of different acetyllysine modifications present on each of the different core and variant histones, bromodomains play a critical role in orchestrating protein and DNA complexes at chromatin. Owing to their central role in chromatin function, bromodomain-containing proteins have been attributed to play prominent roles in the development and progression of a spectrum of diseases, including cancer and other cardiovascular, metabolic, inflammatory, neurologic, and musculoskeletal diseases [23].

**Figure 1:**
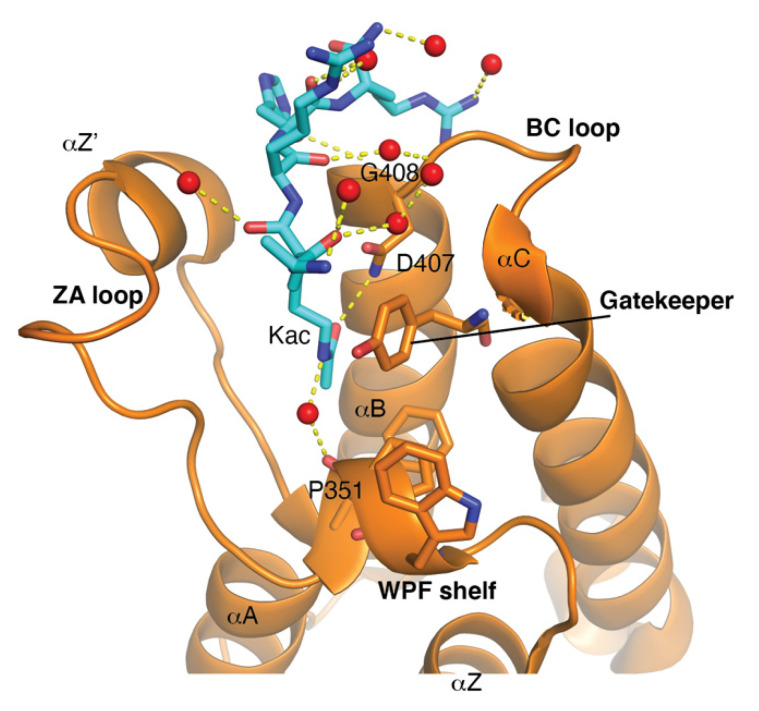
Structural features of the bromodomain binding pocket. The general control non-depressible 5 protein (Gcn5p) bromodomain (orange) is shown in complex with an acetylated histone H4 peptide (cyan) (PDB ID: 1E6I). Hydrogen bonds are indicated by a yellow dotted line, and water is colored in red. This figure was generated with the PyMOL Molecular Graphics System, version 2.4.2, Schrödinger, LLC.

The bromodomain is a 110-amino-acid structural motif that forms of a bundle of four α-helices (αZ, αA, αB, and αC) where the interhelical αZ-αA (ZA) and αB-αC (BC) loops create a hydrophobic pocket that recognizes acetyllysine modifications. Although the primary sequence varies between bromodomains, certain residues in the BC loop region that are involved in Kac coordination are highly conserved [24]. The bromodomain structure from the general control non-depressible 5 (Gcn5p) HAT in complex with an acetylated histone H4 peptide was solved using X-ray crystallography [25]. This structure revealed that the site of Kac recognition was in the hydrophobic pocket formed between the ZA and BC loops. Inside this binding pocket, the carbonyl oxygen on the acetyl group of the lysine forms a hydrogen bond with a nitrogen in the amide group of asparagine 407 in Gcn5p (Figure 1). This asparagine residue is nearly universally conserved in all bromodomains and is important for Kac recognition. Additionally, the histone H4 peptide was found to interact with several ordered water molecules that help stabilize the ligand inside the binding pocket [25].

Bromodomains have been grouped into different subfamilies based on structural similarities. Filippakopoulos et al. provided a structure-based analysis of the human bromodomain family using 34 high-resolution crystal structures and 4 nuclear magnetic resonance (NMR) models, as well as secondary structure predictions, to cluster each bromodomain into eight distinct subfamilies (I–VIII). However, although bromodomains are highly conserved at the structural level, they exhibit relatively low sequence similarity. Of note, the bromodomain module can possess variable lengths of the ZA and BC loop regions, and often have conserved amino acid substitutions in residues in the ligand coordination regions of the binding pocket such as the gatekeeper residue or the WPF shelf (Figure 1) [26]. These important differences in bromodomain structures have allowed for the design of highly selective bromodomain inhibitors [26,27]. The WPF shelf is a distinct hydrophobic part of the acetyllysine binding pocket made up of residues from the ZA loop [28]. These residues (W97, P98, and F99) are conserved in the BET family and are essential for ligand recognition [26]. Inhibitors designed for BET bromodomains are often Kac mimetics that interact with residues in the acetyllysine binding pocket, the WPF shelf, and/or the ZA channel [29]. Another important residue in the bromodomain binding pocket is the so-called gatekeeper residue. This is the first residue in the αC helix, which usually consists of a hydrophobic residue, that can either contain a branched side chain such as isoleucine, or an aromatic Tyr/Phe/Trp. The nature of the gatekeeper residue changes the size, shape, and chemical composition of the bromodomain binding pocket to fine tune the ligand recognition [26].

There are a number of different Lys residues present on the N- terminus of each of the core histone proteins that are known to be acetylated: 10 in histone H2A, 16 in H2B, 13 in H3, and 9 in H4 [30]. The histone ligand binding activity towards specific Kac modifications has been examined in many of the human bromodomains found in different subfamilies [31]. Further characterization of their preferred histone ligands has been carried out using a variety of different biophysical methods that employ recombinant bromodomains and modified histone peptides, or nucleosome substrates in binding reactions [23]. Classically, methods used for ligand identification included Western blots, isothermal titration calorimetry, fluorescent polarization spectroscopy, surface plasmon resonance (SPR), and NMR. Newer, more high-throughput methods include peptide arrays [31,32] and the development of AlphaScreen-based peptide assays to detect bromodomain ligands from many different combinations of histone modifications [33,34]. For example, the ligand binding of the ATPase family AAA domain-containing 2 (ATAD2 and ATAD2B) bromodomains was recently compared using the dCypher assay developed by EpiCypher [34]. The recombinantly expressed GST-tagged bromodomains of the ATAD2 and ATAD2B paralogs were screened against 288 unique histone ligands containing single- and multiple-modified histone peptides representing PTMs found in all four of the core histones. Interestingly, although the ATAD2/B bromodomains are highly conserved and recognize similar histone ligands, it was discovered that the ATAD2B bromodomain has a much broader range of PTM histone binding partners. The ATAD2B bromodomain interacted with 39 ligands from histones H4 and H2A, compared to ATAD2A, which bound to 11 ligands on histone H4. Both of these bromodomains showed a strong preference for histone H4, recognizing acetylated lysine at residues 5, 8, and 12, similar to what was previously reported for ATAD2 using time-resolved fluorescence resonance energy transfer (TR-FRET) methodology [35]. Using isothermal titration calorimetry (ITC) and modified peptides, Lloyd et al. determined the dissociation constants (K_D_) for mono- and di-acetylated histone ligands, demonstrating that the ATAD2B bromodomain preferentially recognizes histone H5K5ac (5.2 ± 1.0 μM), followed by several di-acetylated histone peptides including H4K5acK12ac (18.7 ± 0.9 μM). Other bromodomains have also been shown to recognize di-acetylation modifications, including bromodomain and PHD finger containing 1 (BRPF1), bromodomain containing 9 (BRD9), TATA-box binding protein associated factor 1 (TAF1), and the BD1/BD2 bromodomains in the BET subfamily, suggesting a cooperative role of local sites for ligand binding [31,36,37,38].

Over the last few years, proteomics studies have identified additional acyl modifications found on histone lysine residues that appear at a much lower frequency compared to acetylation [39,40,41]. These include histone lysine propionylation, butyrylation, crotonylation, succinylation, malonylation, 5-hyroxylation, and N-formylation (Kpr, Kbr, Kcr, Ksu, Kmal, Khy, and Kfo, respectively) [39,40,41,42,43]. The bromodomains of BRD4 were shown to recognize Kpr and Kbu, with significantly reduced binding affinities compared to Kac at the same residue [44]. Similarly, 49 human bromodomains were screened for binding to peptides bearing related acyl PTMs, including Kac, Kpr, Kbu, Kcr, Kfo, and Ksu [37]. While Kpr commonly bound to the bromodomains screened, only three bromodomains could bind Kbu (CECR2 histone acetyl-lysine reader, BRD9, and TAF1/L), and a single bromodomain showed binding to Kcr at reduced affinity (TAF1/L) [37]. None of the bromodomains tested showed affinity for Ksu using the peptide array platform. There are also reports of bromodomain interactions with acetylated non-histone proteins, which highlights the complex role that bromodomain proteins play in biology [45,46,47,48]. Lastly, despite the large number of PTMs found on histones, our understanding of how these PTM combinations effect chromatin recognition is very limited. However, this knowledge will be essential for deciphering the comprehensive nature of bromodomain ligand recognition in the context of the epigenetic landscape.

## 3. Expression and Dependency Patterns of Bromodomain Genes across Cancer

The aberrant expression of the genes encoding bromodomain proteins has been frequently associated with a number of different types of cancer, often showing either a favorable or non-favorable prognostic value [49,50,51,52,53,54,55]. Global transcriptomic profiling via high-throughput RNA sequencing (RNA-seq) has proven to be a powerful method for classifying gene expression patterns, identifying biomarkers for disease classification and diagnosis, and uncovering candidate drug targets. The Cancer Genome Atlas (TCGA) and Genotype-Tissue Expression (GTEx) projects have provided RNA-seq data from a large number of cancer and non-cancer samples, providing an exceptional resource for exploring gene expression in cancer research [56,57]. TCGA data are generated across a number of different cancer types. For example, the TCGA has compiled RNA-seq data for over 900 tumor samples of breast cancer (BRCA), with representatives from each breast cancer molecular subtypes, in addition to data for 90 adjacent normal tissues.

**Figure 2:**
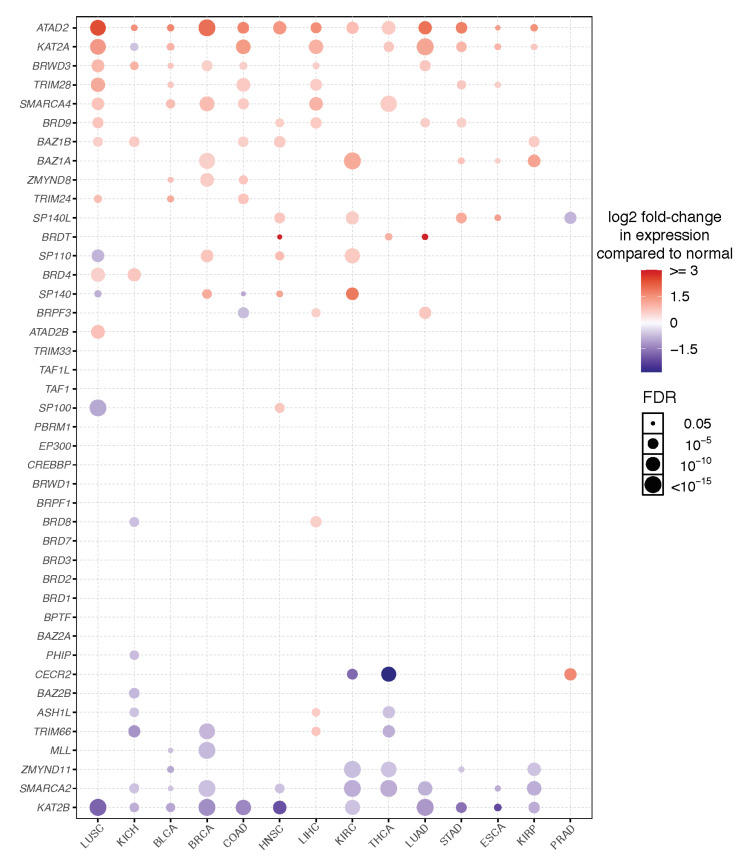
Differential expression patterns of bromodomain-containing protein genes in tumor versus normal tissues for different cancer types. Differential gene expression of bromodomain-containing protein genes comparing tumor and paired normal samples for 14 cancer types in The Cancer Genome Atlas (TCGA) (those with more than ten paired tumor and normal samples). The fold change is mean (Tumor)/mean (Normal), and the *p*-value was determined by a *t*-test adjusted by FDR (false discovery rate). Analysis was performed using the Gene Set Cancer Analysis web server [71].

To summarize the expression patterns of bromodomain genes across different tumor types, the differential expression of bromodomain genes in tumor versus normal tissues are depicted for each of 14 cancer types with adequate numbers of tumor and normal samples (Figure 2). This analysis of gene expression highlights the altered expression patterns of several bromodomain genes across many cancers. A group of bromodomain protein genes are consistently highly expressed across several different tumor types, including *ATAD2, KAT2A, bromodomain and WD repeat domain containing 3 (BRWD3), Tripartite motif 28 (TRIM28), SWI/SNF related, matrix associated actin dependent regulator of chromatin, subfamily A, member 4 (SMARCA4), BRD9,* and *bromodomain adjacent to zinc finger domain 1A (BAZ1A).* The expression of several of these genes has previously been documented in different cancers. For example, the gene encoding the ATAD2 AAA-ATPase bromodomain-containing protein has emerged as a possible therapeutic target in cancer. The *ATAD2* gene has been reported to be overexpressed in a wide variety of cancers, such as endometrial [58], cervical [59], ovarian [60], colorectal [61], and gastric [53] cancers. In breast cancer, *ATAD2* has been identified as an oncogene, and overexpression is an indicator of poor prognosis [62,63,64]. Much less is known about the paralogous *ATAD2B* gene, but it has also been reported to be more highly expressed in several other cancers including brain and breast cancer tumors [65]. Overexpression and/or mutation of the bromodomain-containing KAT2A (GCN5) HAT appears to correlate with aggressive cancer progression and poor prognosis for several different cancers including non-small cell lung cancer, hepatocellular carcinoma, breast, colorectal, and prostate cancers [66,67], and is reviewed in [68]. A number of bromodomain genes are consistently decreased in the TCGA cancer dataset, including *KAT2B, SMARCA2, zinc finger MYND domain-containing protein 11 (ZYMND11),* and *mixed-lineage leukemia (MLL)* [56]. The decreased expression implies tumor suppressor functions for these proteins. For example, decreased *SMARCA2* expression has been documented in several cancer cell lines and primary cancers, and was significantly associated with poor survival of lung cancer patients [69,70]. Overall, this portrait of altered gene expression in the human bromodomain genes highlights both common and unique expression patterns across different cancer types.

A major challenge in cancer research is to identify genes that cancer cells depend on for their growth and survival. Genes that are essential for cell viability in a context-specific manner, as opposed to pan-essential genes, represent potential therapeutic targets. Accordingly, cancer dependency maps generated by genome-scale CRISPR/Cas9 or RNA interference screens have been used to profile the genetic dependencies of diverse cancer cell lines [72,73]. This systematic approach has uncovered many different context-specific dependencies in cancer that could be therapeutically exploited [74,75].

**Figure 3:**
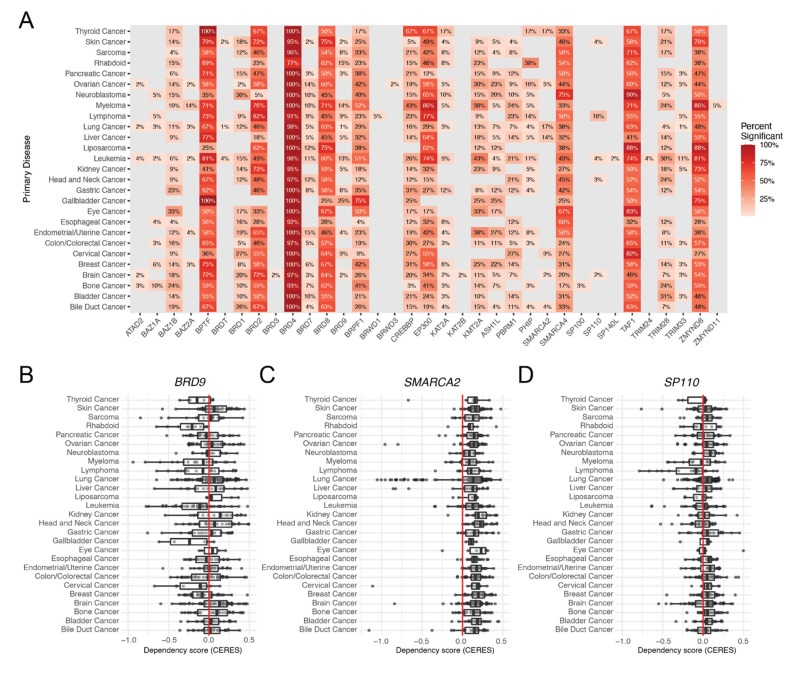
Dependency of bromodomain genes in systematic CRISPR knockout screens from the Cancer Dependency Map project (https://depmap.org/portal/depmap, accessed on 7 July 2021). The CERES score indicates the likelihood that a gene is essential [84]. A CERES score of 0 means the gene is not essential, while −1 is comparable to the median of all pan-essential genes. Data were retrieved from depmap.org (release 21Q1). (**A**) The percentage of cancer cell lines with CERES scores < −0.5 for each BRD gene across disease types. CERES scores for (**B**) *BRD9,* (**C**) *SMARCA2,* and (**D**) *SP110* across disease types.

To examine the dependencies of bromodomain genes across cancer cell lines, we interrogated the dependency scores of bromodomain genes using genome-scale loss-of-function screens available through the cancer dependency map (DepMap) project (Figure 3A) [76]. This analysis reveals that certain bromodomain genes are essential across cancer cell lines derived from different tissue types, while other bromodomain genes exhibit low to no essentiality in cancer cell lines. For example, *BRD4* exhibits a high percentage of essentiality across nearly all cell lines for a given tissue (near 100%). *BRD4* has been classified as an ‘pan-essential gene’ according to the DepMap project [76]. Interestingly, several bromodomain genes exhibit context dependency patterns in a subset of cell lines, and may represent a subtype that is vulnerable to bromodomain targeting therapies. In contrast, *BRD9* is a bromodomain gene that shows variable dependency scores across different cancer cells (Figure 3B). Interestingly, *BRD9* has recently been characterized as an essential gene in the pediatric rhabdoid tumors [77]. Malignant rhabdoid tumors (MRT) are a highly aggressive cancer driven by truncating mutations in the *SMARCB1* gene encoding a subunit of the BAF chromatin-remodeling complex [78,79]. This specific dependence upon a *BRD9* was shown to occur through a unique BRD9-SWI/SNF subcomplex distinct from BAF and PBAF complexes that lacks SMARCB1 [80]. Both RNA interference (RNAi)-mediated *BRD9* knockdown and CRISPR-Cas9-mediated *BRD9* knockout compromised the proliferation of SMARCB1-mutant MRT cells, providing compelling support that BRD9 is a therapeutic target for *SMARCB1*-mutated cancers [81,82]. SMARCB1-mutant MRT cells were unresponsive to recently developed BRD9 bromodomain inhibitors, which were shown to have efficacy in leukemia cells [80]. However, Wang et al. showed that the previously uncharacterized DUF3512 domain of BRD9 mediates SWI/SNF complexes in MRT [77]. The SMARCA2 gene also demonstrates context-dependent vulnerabilities in several tissue types including lung cancers (Figure 3C). SMARCA2 has been identified as an essential gene in lung cancer cells that harbor SMARCA4 mutations [83]. Additionally, 18% of lymphoma cell lines are significant for *SP110* depletion, which may reveal a population of lymphoma subtypes that are vulnerable to prospective SP110 inhibitors (Figure 3D). Thus, cancer gene vulnerability analysis provides valuable information for understanding the potential for targeting different bromodomain proteins in various cancer types.

## 4. An Interconnected Network of Functional Groups in Bromodomain Proteins

**Figure 4:**
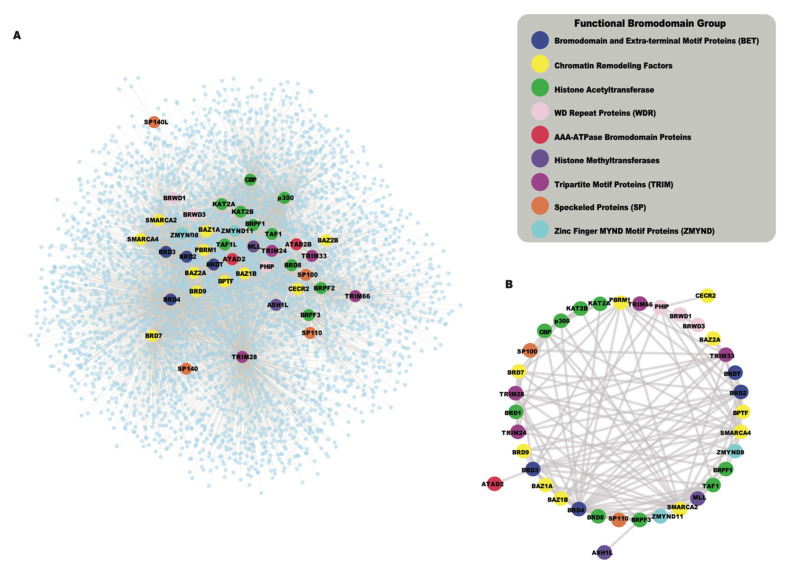
A highly connected functional bromodomain protein-protein interaction (PPI) network. (**A**) Interaction network of bromodomain-containing proteins. The public PPI datasets contain curated PPI derived from a range of affinity purification approaches including affinity capture followed by mass spectrometry (MS), as well as proximity labelling and MS, yeast two-hybrid methods, and others [91]. Accordingly, all physical interactions for the 42 bromodomain-containing genes were processed and plotted with Cytoscape. Each of the bromodomain-containing proteins are displayed in a larger size, and are color coded by their respective functional group as indicated in the figure legend. (**B**) Interactions between different bromodomain-containing proteins. Interactions between 37 bromodomain proteins are depicted, as 5 bromodomain proteins do not interact with other bromodomain-containing proteins.

While the human bromodomains have been divided into eight subfamilies based on their structural features, they are found in a wide range of proteins with diverse catalytic and scaffolding functions. Bromodomains often act in concert with other functional modules present in the same proteins, and in their associated proteins complexes. As bromodomain-containing proteins assemble into larger complexes to confer context-specific activity, the systematic analysis of protein–protein interactions (PPIs) is useful to summarize potential functional pathways associated with the cellular roles of these chromatin reader proteins. The availability of high-throughput proteomic techniques, such as Affinity Purification coupled to Mass Spectrometry (AP-MS) [85], BioID [86], and recently developed TurboID [87] enabled us to collect protein–protein interaction data from public databases including the Biological General Repository for Interaction Datasets (BioGRID) [88] and the Human Integrated Protein–Protein Interaction reference (HIPPIE) [89], as well as the newly reported interactions for BETs [45]. The global interaction network of all human bromodomain proteins collected from the above resources is shown in Figure 4A, and listed in Table S1. This interaction network shows a highly interconnected map of PPIs of bromodomain proteins grouped according to their function-based classifications. Different functional classes of bromodomain proteins cluster together in the network, based on shared interactions, and the formation of related functional complexes. For example, the BET family of bromodomain proteins, including BRD2, BRD3, BRD4, and BRDT (Bromodomain Testis associated) shown in blue, cluster together as they are well known to share many common interaction partners [45,90]. Similarly, the HAT enzymes including the p300 and cyclic AMP response element-binding protein (p300/CBP), and the monocytic leukemia zinc-finger protein and MOZ-related factor (MOZ/MORF) groups cluster together in this network. Additionally, we also identified a subnetwork of bromodomain–bromodomain protein interactions that demonstrates how the bromodomain-containing proteins interact with each other (Figure 4B) Thus, functional clustering based on their PPI networks provides a succinct way to represent the many inter-connected roles for bromodomain-containing proteins. Below, we discuss nine different functional groups of bromodomain proteins, highlighting recent insights into bromodomain-specific mechanisms in cancer development.

### 4.1. BET Family of Bromodomain Proteins

One of the best-characterized classes of bromodomain-containing proteins is the BET family (BRD2, BRD3, BRD4, and BRDT). The BET family of proteins have been primarily reported to be involved with transcriptional regulation, with defined roles in cellular proliferation and differentiation. BET proteins function to regulate transcription through a variety of mechanisms involving protein–protein interactions with acetylated histones, transcription factors, and chromatin remodeling factors. Each protein within the BET family contains two tandem bromodomains at their amino-terminus as well as a conserved extra-terminal (ET) domain at the carboxy-terminus. The ET domain consists of approximately 80 amino acids, and this region is known to interact with several chromatin effector proteins, including Jumonji Domain Containing 6 (JMJD6), Chromodomain Helicase DNA binding Protein 4 (CHD4), Glioma Tumor Suppressor Candidate Region 1 (GLTSCR1), Nuclear Receptor Binding SET Domain Protein 3 (NSD3), and ATPase Family AAA Domain Containing 5 (ATAD5) [92]. In addition, BRD4 and BRDT (but not BRD2 and BRD3) possess a C-terminal domain (CTD) that interacts with the Positive Transcription Elongation Factor b (P-TEFb) [93]. This complex phosphorylates serine residues of the CTD of RNA polymerase II to promote transcriptional elongation [94,95]. BRD4 recruits P-TEFb to hyperacetylated genomic regions, including Transcription Start Sites (TSS) and clustered enhancers (called super-enhancers) [96,97]. BRD4 interacts with the Mediator complex to promote target gene transcription [98,99]. The BRDT CTD also interacts with P-TEFb, but BRDT expression is restricted to the testis where it participates in gene expression and splicing in spermatogenesis [100,101,102].

BET bromodomains exhibit a preference for closely spaced di-acetylated Lys residues on their histone targets. The first bromodomain of the tandem pair exhibits a preference for acetylated lysine residues in histone H4, with a higher affinity for H4K5ac, whereas the second bromodomain binds more promiscuously to different acetyllysine residues [38]. The co-crystal structure of the modified histone H4K5acK8ac peptide bound to the first bromodomain of BRD4 revealed that H4K5ac is recognized through the canonical mode of acetyllysine binding, which is mediated through hydrogen bonds with the conserved Asn140 and Tyr97 residues. The adjacent H4K8ac forms hydrophobic interactions with Trp81 to fortify H4K5ac recognition [31].

The discovery of JQ1 as a highly specific and potent small-molecule inhibitor of the BET bromodomains, with little activity toward non-BET bromodomain, rapidly stimulated a global interest in drug discovery efforts targeting this family of bromodomain-containing proteins [27]. Notably, pharmacological inhibition of BET bromodomains has broadly been shown to suppress oncogenic gene expression programs, reducing the expression of oncogenes including c-Myc across many blood and solid tumor cancer types [103,104]. Inhibition of BRD4 results in a selective decrease in transcription at super-enhancer associated genes as a result of the dissociation of BRD4, Mediator, and P-TEFb. Thus, BET inhibitors have shown promising results in tumors that are dependent on these transcriptional programs including castration-resistant prostate cancer [105], breast cancer [106], non-small cell lung cancer, gastrointestinal cancers including colon cancer, gliomas, and several hematological malignancies such as acute myeloid leukemia [107], lymphoma, and multiple myeloma [108]. The development of new BET inhibitor (BETi) compounds, which now includes potent inhibitors for individual BET proteins [109], and those targeting specific bromodomains (either BD1 or BD2) in the BET family [110,111], has generated an immense amount of new knowledge about the physiological roles of BRD2/3/4/T.

More recently, a systematic proteomic approach was used to analyze the overall protein interactions of BET proteins. Lambert et al. carried out affinity purification on the BET bromodomains followed by mass spectrometry (AP-MS) before and after the addition of the pan-BET inhibitor JQ1 [45]. Quantitative analysis of 603 unique interacting proteins defined three distinct sets of BET protein interactions including those that occur through the canonical acetyllysine binding pocket to recognize acetylated histone and non-histone proteins, as well as acetylation-independent interactions via the extra-terminal domain. This study examined the human proteome to identify di-acetyllysine motifs on histone and non-histone proteins, and further characterized the interaction of BET bromodomains with these Kac-XX-Kac motifs using a combination of biophysical, structural, and cell biology approaches. Importantly, they identified several new non-histone interactions, some that were increased upon addition of JQ1, and found the ET domain provides an important protein recruitment platform. They also demonstrated that BRD3 is a negative regulator of cellular proliferation via regulation of ribosomal RNA production. This comprehensive study is the first to clearly demonstrate how small-molecule inhibitors targeting specific bromodomain containing proteins can be effectively used as tool compounds to elucidate the biological functions of bromodomains as well as inhibitor action.

### 4.2. Chromatin Remodeling Factors

An additional group of bromodomain-containing proteins have central roles in chromatin remodeling, functioning directly in enzyme catalysis, or as regulatory subunits in chromatin remodeling complexes. Chromatin remodeling controls the higher order structures of DNA and regulates the accessibility of specific genomic elements [112]. This activity is mediated by large protein complexes that coordinate modulation of the chromatin conformation to regulate a variety of biological processes including transcription, recombination, DNA repair, and DNA replication. The mammalian SWItch/Sucrose Non-Fermentable (mSWI/SNF) complexes are an important group of ATP-dependent chromatin remodeling complexes that contain a bromodomain module. These include SMARCA2 (which is also known as BRM for brahma homologue), SMARCA4 (BRG1, for Brahma-related gene-1), BRD7, BRD9, and Polybromo 1/BRG1-Associated Factor 180 (PBRM1/BAF180) [113,114]. The mSWI/SNFs are grouped into three distinct complexes: The canonical ATPase BRG1/BRM-associated factor (cBAF), the polybromo-associated BAF (PBAF), and a recently defined non-canonical BAF (ncBAF) complex [115]. Each of these complexes contain an anchoring ATPase and bromodomain (SMARCA2/4), as well as additional bromodomain-containing subunits [116]. BRD7 is a component of the polybromo-associated BRG1-associated factor (PBAF)-specific SWI/SNF chromatin remodeling complexes, while the ncBAF complex was shown to contain BRD9 [117,118].

The ability of the bromodomain to bind Kac likely contributes to numerous aspects of mSWI/SNF function. For example, the assembly of specific SWI/SNF complexes at target genomic regions is believed to be determined in part via interactions with the SMARCA2/SMARCA4 bromodomain and acetylated chromatin [119]. Both bromodomains of SMARCA2/SMARCA4 have been characterized to bind preferentially to histone H3 acetylated at lysine 14 (H3K14ac) in vitro [119,120]. A recent study identified an AT-hook motif adjacent to the bromodomain in SMARCA2/SMARCA4 that binds to double-stranded DNA in vitro [121]. AT-hooks are arginine/lysine-rich and contain a central glycine-arginine-proline (GRP) sequence that allows the motif to be inserted into the minor groove of DNA at AT-rich elements [122]. A follow up study used SELEX-seq (systematic evolution of ligands by exponential enrichment sequencing) to identify a preferential A/T-rich DNA consensus site for the SMARCA2/SMARCA4 bromodomain AT-hook region in vitro [123]. AT-hook sequences can be found in several other chromatin reader proteins alongside chromodomains and PWWP domain-containing proteins [124]. Thus, a multivalent mode of chromatin recognition involving histone Kac binding and DNA recognition may be a common mode for bromodomain function. Notably, small-molecule inhibition of the SMARCA2/4 bromodomains does not decrease their chromatin association in cells, unless pre-treated with HDAC inhibitors [125,126,127]. Additional investigation needs to be performed to evaluate how the AT-hook bromodomain contributes to the SMARCA2/4 chromatin interaction in various contexts. SMARCA2/4 are known to be regulated by post-translational modification including phosphorylation and acetylation [128,129], which could potentially regulate chromatin interactions through mechanisms involving DNA recognition.

SMARCA2/4 (BRM/BRG) have been widely studied; SMARCA4 is frequently mutated in cancer and maintains oncogenic transcription and cellular proliferation in acute myeloid leukemia (AML) [130]. However, SMARCA4 also has tumor suppressor activities in solid tumors, similarly to SMARCA2, which is generally classified as a tumor suppressor [130]. Thus, the loss of BRM and BRG in humans results in the dysregulation of genes associated with lung cancer development and progression, leading to an overall tumorigenic phenotype [131]. Interestingly, SMARCA4 expression is correlated with liver hepatocellular carcinoma and kidney renal clear cell carcinoma, while overexpression of SMARCA2 is associated with better patient outcomes [132]. Due to its long-standing connection to cancer development, and the fact that the BRM/BRG complexes control the expression of many cancer-associated genes, there have been several recent studies that report the use of small-molecule inhibitors to target SMARCA2/4 bromodomains. For example, bromodomain inhibitors specifically targeting the SMARCA2 and SMARCA4 BRDs bound in the nanomolar range and were able to prevent acetyllysine recognition [127]. An AlphaScreen-based assay used to screen a large compound library also identified a SMARCA2 BRD inhibitor known as DCSM06, which may provide new information about the tumor suppressor functions of this protein [133]. However, these inhibitors were either not specific enough for SMARCA4, or they did not produce the desired anti-proliferative effects.

The mammalian Imitation Switch (ISWI) ATP-dependent chromatin remodeling complexes are comprised of an ATPase subunit (SNF2L and SNF2H) in complex with different regulatory subunits consisting of various bromodomain-containing proteins, including BAZ1A (ACF1), BAZ1B (WSTF), BAZ2A (TIP5), BAZ2B, Bromodomain PHD finger Transcription Factor (BPTF), and CECR2 [134]. For example, the association of SNF2L and BPTF makes up the nucleosome remodeling factor (NURF) complex, which is the founding member of the ISWI family of chromatin remodelers [135,136,137]. NURF plays an important role in transcriptional regulation and functions by remodeling a higher-order chromatin structure downstream of various signal transduction pathways (reviewed in [138]). The histone ligands for BPTF have recently been characterized, and the bromodomain associates with acetyllysine modifications in the histone variants H2A.Z I and H2A.Z II, preferentially binding to H2A.Z II di-acetylated at lysine 7 and 13 [139]. Similarly, this study also demonstrated that the bromodomain of CECR2 binds acetylated H2A.Z isoform I. These results suggest that the distinct transcriptional programs of the NURF or CERF (CECR2-containing remodeling factor) ISWI chromatin remodeling complexes are mediated by the acetylation of different histone variants. The fundamental outcomes of these histone ligand recognition events require further study; however, structural studies on the BPTF bromodomain should aid in the further development of selective and potent BPTF bromodomain inhibitors, which will further dissect the functionalities of ISWI complexes in chromatin remodeling [140].

The ISWI subunit BAZ1A was recently discovered as a regulator of cellular senescence [141], and the related BAZ2A subunit is also known to be overexpressed in prostate cancer, where it may be involved in prostate cancer metastatic regulation [142]. As such, BAZ protein expression could be utilized as a prognostic marker. Recently, a BAZ1A inhibitor called Cpd-2 was discovered with a potency of 520 nM [143]. Additionally, the inhibitor GSK2801, developed by GlaxoSmithKline, has been shown to dually inhibit BRD9 and BAZ2A/B, and act synergistically with BET inhibitors to induce apoptosis in triple negative breast cancer (TNBC) cells [144]. While BAZ2B has a less well-defined function than its paralogue BAZ2A, inhibitors also exist that are selective for BAZ2A/B over other BRDs in this family. For example, the BAZ-ICR inhibitor was shown to act as a selective and potent dual inhibitor for the BAZ2A/B isoforms that can be utilized in cellular assays to further probe the function of BAZ2 bromodomains [145].

Much less is known about SWI/SNF subunits BRD7 and BRD9 when it comes to their roles in cancer. BRD7 has recently been found to act as a tumor suppressor gene, and its expression is down-regulated in cancers like breast cancer, nasopharyngeal carcinoma, prostate cancer, and ovarian cancer [146]. BRD7 has been shown to regulate several cellular signaling pathways by interacting directly with p53 to prevent cellular proliferation, and with BRCA1 to regulate transcription of the estrogen receptor alpha [147,148]. On the other hand, BRD9 appears to have some oncogenic properties as the inhibition of BRD9 induces apoptosis in TNBC and blocks cellular proliferation in AML [80,144]. Thus, the opposing functionalities of the closely related BRD7 and BRD9 proteins, as well as in SMARCA2/4, highlights the importance of understanding the roles of specific bromodomain-containing proteins in cancer biology in order to effectively develop new therapeutic strategies.

### 4.3. HAT Bromodomain Proteins

Another well-characterized class of bromodomain-containing proteins is the group that either possesses intrinsic histone acetyltransferases activity or associates with HAT complexes as transcriptional coactivators. The bromodomain is thought to anchor the HAT complex at acetylated chromatin, allowing it to further acetylate adjacent nucleosomes, or regulate the assembly of transcriptional complexes through protein–protein interactions [149]. Therefore, the role of the bromodomain module in the HAT complex likely enhances the histone acetylation signals through the recruitment of HATs at pre-acetylated nucleosomes. Interestingly, the HATs GCN5 (KAT2A), PCAF (KAT2B), CBP (KAT3A also known as *CREBBP*), and p300 (KAT3B or *EP300*), which all contain an intrinsic bromodomain, were also found to cluster together in the PPI network (Figure 4A, shown in green). These enzymes share many protein substrates, including histones, transcription factors, nuclear receptors, and enzymes, playing central roles in the positive regulation of transcription [150,151].

Like BRD4, the bromodomain-containing HAT/KAT proteins are known to be associated with enhancers; however, the role that the bromodomain plays in connecting each HAT with its associated cellular function(s), is not completely clear. A recent study investigated the bromodomain-associated mechanisms regulating the function of the p300/CBP HAT. A p300 protein lacking the bromodomain region was used to demonstrate that the loss of this domain prevented p300 from maintaining the basal level of histone acetylation [152]. Moreover, using the CBP bromodomain-specific inhibitor GNE-049, Raisner et al. reported a reduction of H3K27ac enrichment at the enhancers, without a major loss of CBP/p300 occupancy at these elements in chromatin [153]. Similarly, combination treatment of the small molecule bromodomain inhibitor (I-CBP112) with the p300/CBP active site inhibitor (A-485) resulted in a dramatic reduction in p300 chromatin enrichment, and impaired the expression of androgen-dependent and pro-oncogenic genes including *KLK3* (encoding PSA) and *MYC*, compared to the individual effects of blocking each domain alone [154]. CBP/p300 have been widely implicated in cancers, specifically hematological malignancies, due to their role in transcriptional regulation of hematopoiesis. Disruption or depletion of p300/CBP leads to defects in normal hematopoiesis and may aid cancer progression [155]. Due to their strong association with cancer, the development of inhibitors targeting the CBP/p300 BRD and HATs have been at the forefront of BRD-associated inhibitor development for the past decade [156]. Importantly, one highly promising inhibitor, CCS1477, is currently undergoing clinical trials for the treatment of hematological malignancies and advanced prostate cancer (https://clinicaltrials.gov/ct2/show/NCT03568656, accessed on 7 July 2021).

Another group of HAT complexes known as the MYST family include Tip60, MOZ, MORF, HBO1 (Histone acetyltransferase Binding to ORC1), and MOF (Males absent On the First). These HATs generally contain multiple subunits in addition to the catalytic MYST domain responsible for acetylating histone substrates [157]. BRPF1 is part of the MOZ/MORF HAT complex, which dictates the acetylation of all four core histones, and is actively involved in chromatin remodeling [158,159]. MOZ was first identified in chromosomal translocations associated with acute myeloid leukemia [160,161,162,163]. BRPF1 functions to stimulate the catalytic activity of the MOZ/MORF complex and also interacts with the inhibitor of growth 5 (ING5) subunit in the complex [164]. The recognition of acetyllysine by the BRPF1 bromodomain is thought to bridge the MOZ/MORF complexes to chromatin, promoting further acetylation of nearby histones to increase transcription in those areas [149]. BRPF1 inhibitors have been developed that select for BRPF1 against other family IV BRDs. The selective inhibitor IACS-9571 has dual specificity towards the BRPF1 and TRIM24 bromodomains and may be therapeutically useful for acute myeloid leukemia or breast cancer patients [165]. Meanwhile, the recent development of more specific BRPF1 bromodomain inhibitors was shown to hinder cellular viability in a leukemia cell line [166]. Interestingly, another inhibitor that was developed targeting the BRFP1B isoform (OF-1) demonstrated cellular activity in the regulation of osteoclastogenesis, which may provide new strategies to prevent bone loss or bone-related malignancies [167].

BRPF2 and BRPF3 are part of the HBO1 HAT complex, which primarily acetylates histone H4 and helps to facilitate gene transcription through the control of chromatin dynamics [168]. BRPF2 has been shown to directly regulate the HAT activity of the HBO complex, acting in a similar fashion as BRPF1 with regards to its interaction with MOZ [169]. Although both BRPF2 and BRPF3 are components of the HBO1 HAT complex, they appear to alter the functionality of this HAT. BRPF2 has been shown to be essential for the global acetylation of H3K14ac and serves to activate transcriptional programs for erythroid development [170]. An inhibitor selective for BRPF2 and the TAF1 and TAF1L proteins, BAY-299, was shown to prevent specific histone interactions, and would likely re-direct the acetylation activity of the associated HAT complexes [171]. Meanwhile, the function of BRPF3 has been associated with the recognition of H3K14ac modifications enriched at DNA origins of replication, broadly implicating BRPF3 as an essential player in regulating the initiation of DNA replication [172]. BRPF3 overexpression causes an upregulation of the KAT7 HAT complex, which leads to the dysregulation of embryonic development and cell cycle progression, suggesting that it may be an important contributor to cancer development [173]. Taken together, the bromodomain proteins within the HAT complexes appear to play important roles in the recognition of acetylated chromatin. They function to finetune different cellular outcomes by bridging the engagement of specific HATs at the promoters of actively transcribed genes, upregulating the HAT activity to promote acetylation of lysine residues within the histone tails, and play an important role in maintaining cellular levels of acetylation.

### 4.4. WD Repeat Proteins

Another family of bromodomain-containing proteins is the WD-repeat (WDR or WD-40) family. This family consists of the proteins WDR9 (WD repeat protein 9 or BRWD1), BRWD3 (Bromodomain and WD-repeat containing protein 3), and PHIP (pleckstrin homology domain-interacting protein). WDRs are comprised of a broad variety of proteins that all contain a beta-propellor-shaped WD repeat domain [174]. WDRs are involved in a wide variety of cellular functions including epigenetic regulation, DNA damage repair, and cell cycle regulation [175]. WDRs can also act as versatile protein scaffolds and some are considered promiscuous interactors [176]. A subgroup of the WD-repeat family was found to be among the top 10 ranked protein–protein interactors, and are one of the most abundant domains in the human proteome [176]. In the last few years, WDRs have emerged as promising drug targets due to their broad network of protein–protein interactors, and their implications in the epigenetic basis of cancer [177]. The WD-repeat proteins BRWD1, BRWD3, and PHIP also contain a bromodomain.

BRWD1 has several functions in regulating the immune system. BRWD1 is recruited to the immunoglobulin kappa (IgK) locus via epigenetic signals where it enhances RAG recruitment to help facilitate B cell recombination [178]. By controlling enhancer accessibility, BRWD1 has also been shown to regulate over 7000 genes to facilitate B cell differentiation and cell growth [179]. While the function of BRWD3 in relation to cancer development is unknown at this time, BRWD3 was shown to be up-regulated in breast cancer patient’s plasma and has potential for use as a serological biomarker [180]. Another WDR family member, PHIP, has been shown to promote the growth of breast cancer, lung cancer, and melanoma tumor cells [181]. PHIP suppression can significantly inhibit tumor cell invasion and progression. PHIP downregulation coincided with the suppression of AKT phosphorylation, cyclin D1 expression, and talin1 expression in all types of tumors. The bromodomain of PHIP binds to the histone H4K91ac epigenetic mark providing a functional role for PHIP’s bromodomain and presented it as a drug target with therapeutic potential against these tough-to-treat tumor types that lack specific molecular drivers [181]. Additionally, the increased expression of PHIP was correlated with a marked decrease in the overall survival rate in HER2+ breast cancer tumors [55]. A recent review highlights the potential drugability of WDR family proteins and their potential use as oncological targets [177]. The WDR family of bromodomains is an understudied group with potential implications in a variety of diseases and developmental disorders. Thus, they display promise as potential therapeutic targets by modulating either the WDR or bromodomain interactions.

### 4.5. AAA-ATPase Bromodomain Proteins

The AAA-ATPase bromodomain proteins ATAD2 (ANCCA) and ATAD2B are highly related paralogs that contain two AAA-ATPase domains (ATPase associated with diverse cellular activities), and a C-terminal bromodomain. The ATPase domain has been shown to be important for the assembly of oligomeric complexes [182], while the bromodomain is known to recognize acetylated histones [183,184]. ATAD2 interacts with the MYC oncogene and stimulates transcriptional mediated cell proliferation. Therefore, it has the potential to contribute to more aggressive cancers through MYC-dependent proliferation [62]. ATAD2 was also shown to be a co-activator of the estrogen and androgen receptors [185,186]. In cell proliferation, ATAD2 initiates and sustains a transcriptional positive feedback loop up-regulating itself and target genes [187]. ATAD2 is overexpressed in multiple types of cancer including breast, lung, gastric, endometrial, colorectal, renal, and prostate [51,52,53,63,64,185,188,189,190], and overexpression of ATAD2 is often correlated with poor patient outcomes, and can be used as prognostic marker [51,52,53]. In addition to its role as a driver of cellular proliferation, ATAD2 function has also been linked to DNA repair and a higher-order chromatin structure [35]. Koo et al. demonstrated that ATAD2 expression is linked to the S-phase and is localized at sites of DNA replication. Furthermore, ATAD2 was associated with newly synthesized histone H4K5acK12ac modifications that occur immediately following replication [35]. This finding supports previous research showing the bromodomain of ATAD2 functions to recognize H4K5ac and H4K12ac modifications [183,184]. Interestingly, the presence or absence of a disulfide bridge located at the bottom of the bromodomain binding pocket influences ligand recognition of ATAD2, thus acetyllysine binding could be tied to the redox status of the cell [191]. In addition, it appears that the ATPase domain plays an important role in chromatin recruitment, as the bromodomain alone was not sufficient. As such, ATAD2 likely utilizes multivalent interactions with the ATPase and bromodomains to facilitate interactions with the nucleosome that regulate the formation of higher-order chromatin structures [35].

Since ATAD2 overexpression is correlated with cancer development, several inhibitors have been developed to target the ATAD2 bromodomain. GlaxoSmithKline used a fragment-based approach to develop initial inhibitors for the ATAD2 bromodomain, which were later optimized using structural information into highly potent and selective inhibitors [192,193]. Further development of these compounds produced cell permeable inhibitors, but unfortunately, GSK8814 showed only weak in vivo antiproliferative effects [194]. The novel ATAD2 bromodomain inhibitor AM879 was effective in preventing cell proliferation, and induced apoptosis in TNBC cells. Additionally, it also suppressed c-Myc expression leading to the conclusion that ATAD2 plays a role in the expression of c-Myc [195].

ATAD2B is a lesser studied, but highly conserved, paralogue of ATAD2. Recently, the ATAD2B bromodomain was shown to recognize both mono- and di-acylated histone tails with low micromolar affinity, specifically on histones H2A and H4 [34]. Similar to ATAD2, the ATAD2B bromodomain preferentially recognizes H4K5ac and the di-acetylated H4K5acK12ac ligands. However, the ATAD2B bromodomain has a broader histone binding specificity, binding to 39 different post-translationally modified histone ligands, while the ATAD2 bromodomain selected for 11. In addition, the ATAD2B bromodomain binding activity is uniquely regulated through an alternative splice site not found in ATAD2 [34]. Despite these differences, the ATAD2 and ATAD2B bromodomain binding pockets share extensive structural overlap. Thus, it is not surprising that the ATAD2 inhibitor Compound 38 developed by GlaxoSmithKline [193] was found to bind the ATAD2B bromodomain using a conserved set of residues as in ATAD2. These data suggest that many ATAD2 inhibitors are likely interchangeable in selecting for both ATAD2 and ATAD2B [34]. However, an isoform-selective inhibitor targeting only the ATAD2 bromodomain was successfully developed by Bayer [196]. BAY-850 impairs ATAD2 from associating with chromatin through the impairment of the bromodomain by inducing bromodomain dimerization. The development of novel inhibitors selectively targeting the ATAD2 and ATAD2B bromodomains has been essential in identifying new functions for these proteins, and their contributions to cancer development. It is likely that new therapeutic strategies will emerge as we learn more about the epigenetic signaling programs regulating their cellular activities.

### 4.6. HMT Enzymes

Bromodomain-containing proteins have an intimate interaction with a variety of epigenetic marks on chromatin. Methylation is the most prevalent epigenetic modification present in the human genome. Two of the human histone methyltransferases (HMT) also contain a bromodomain, namely ASH1L (absent, small, or homeotic-like protein) and MLL. While the function of the bromodomain within these two HMTs is not well understood, MLL has been studied extensively since chromosomal translocations of the *MLL* gene are well known to be associated with leukemia development [197,198]. The *MLL* gene codes for a histone methyltransferase enzyme that writes H3K4me3, and acts as a transcriptional co-activator, upregulating gene expression. One of the main functions of MLL is an epigenetic regulator important for the maintenance of *Homeobox* (*Hox)* gene expression levels, which direct body segment development [199]. MLL assembles into a large multiprotein complex that acts as a transcriptional activator, and forms a core complex with three additional structural proteins; Retinoblastoma-Binding Protein 5 (RbBP5), ASH2L, and WDR5, which regulate its methyltransferase activity [200]. The transcriptional activation domain of MLL was also shown to interact with the CREB binding protein (CBP) complex, which also functions as a transcriptional activator through its HAT activity [201]. MLL contains four PHD fingers and an atypical bromodomain module that lacks the conserved asparagine usually involved in acetyllysine coordination. It was shown that instead of recognizing acetyllysine, the bromodomain of MLL enhances the interaction of the adjacent PHD3 domain with H3K4me3 [202]. Chromosomal translocations between the *MLL* and *CBP* genes result in a fusion protein that has lost the MLL SET (Su(var), E(z), and Trithorax) domain, and gained HAT activity along with a bromodomain region [203]. It is thought that the aberrant acetylation activity of this complex is a driver of leukemogenesis. More recently, MLL fusion proteins were shown to interact with the BET bromodomain proteins through their interaction with the super elongation complex. As such, small-molecule inhibitors targeting the BET family may be a potential therapeutic approach for these aggressive leukemias [90]. However, another study using BET inhibitors to target leukemias induced by an MLL-AF9 fusion protein found that resistance emerges due to increased expression of Wnt/beta-catenin, presenting possible limitations regarding the use of BET inhibitors to treat MLL-associated leukemias [204].

The ASH1L protein is another bromodomain-containing protein that functions as a HMT. A study looking at the mRNA levels of bromodomain-containing proteins in breast cancer showed that high levels of ASH1L are correlated with poor overall survival rates, but little is known about the functional role the bromodomain [55]. Moreover, there appears to be some functional overlap between ASH1L and MLL in leukemia pathogenesis. Zhu et al. showed that ASH1L writes histone H3K36me2, an epigenetic mark associated with increased transcriptional activity. This modification is bound by the LEDGF protein, which promotes association of the MLL complex with leukemia target genes [205]. Thus, both of these two bromodomain-containing methyltransferases appear to contribute to leukemia development, and ASH1L may provide a new avenue for targeted treatment strategies.

### 4.7. TRIM Family of Bromodomain Proteins

Tripartite motif (TRIM) proteins belong to a large E3 ligase family of proteins that function in diverse cellular processes including apoptosis, cell cycle, cell proliferation, oncogenesis, and viral responses [206,207]. TRIM proteins are characterized by the presence of a conserved N-terminal tripartite motif that consists of a RING (Really Interesting New Gene) domain, B-box zinc fingers, and a coiled-coil region [206]. A group of four TRIM proteins, including TRIM24 (TIF1α), TRIM28 (TIF1β or KAP1), TRIM33 (TIF1γ), and TRIM66 (TIF1δ), make up the transcriptional intermediary factor (TIF1) family proteins because they also contain a C-terminal PHD finger and a bromodomain in tandem [208,209]. Interestingly, the RING domain of TRIM24, TRIM28, and TRIM33 does not appear to confer E3 ligase activity as the isolated domains were unable to ubiquitinylate free lysines of the target proteins to tag them for degradation [210]. However, these proteins have been shown to function as E3 ligases in cellular assays, which is likely attributed to their association with other proteins to confer this activity [211]. Notably, TRIM66 lacks the RING domain, but does contain the B-box, coiled-coil region, and the PHD-bromodomain. The TIF1 family proteins are important regulators of many cellular processes including heterochromatin formation, DNA repair, and genomic integrity.

TRIM24 was first linked to cancer development through its role as an important regulator of p53 [212]. TRIM24 was shown to be a binding partner of p53 through mass spectrometry and co-immunoprecipitation experiments. The inhibition of TRIM24 by RNAi resulted in increased levels of p53 in the nucleus of embryonic stem cells. TRIM24 is known to be highly overexpressed in several cancers including non-small cell lung cancer [213], breast cancer [214], cervical cancer [215], hepatocellular carcinoma [216], as well as prostate [217] and gastric cancers [218]. TRIM24 was shown to target p53 for ubiquitination and subsequent degradation via its RING domain [212]. Thus, overexpression of TRIM24 in cancer is thought to negatively regulate p53, resulting in a loss of its tumor suppressor activity. The PHD-Bromodomain module of TRIM24 has been shown to function as a chromatin reader domain responsible for the recognition of histone H3 that was unmodified, methylated at lysine 9 (H3K9me), or acetylated at lysine 9 and 14 (H3K9ac/K14ac) [212]. Furthermore, the recognition of these modifications link TRIM24 binding to estrogen response elements, where it activates the transcription of the estrogen receptor alpha (ERα) and downstream genes, contributing to cancer development [212]. Bromodomain-specific inhibitors were developed for TRIM24 as a potential cancer treatment [165]; however, targeting the bromodomain alone does not always prevent chromatin binding or cellular proliferation [219].

TRIM28 was discovered as a co-repressor of the Kruppel-associated box (KRAB) domain, which functions as a repressive DNA binding domain found in many transcription factors [220]. TRIM28 works synergistically by binding to the KRAB repression domain through its B box and coiled-coil region to repress transcription [221]. Both TRIM24 and TRIM28 have been shown to interact with the heterochromatin protein 1 (HP1) family through an HP1 box, and they possess intrinsic kinase activity, phosphorylating themselves and HP1 proteins [222]. It has been proposed that one mechanism driving gene repression by TRIM28 occurs through the recruitment of TRIM28 to specific chromatin loci through its interaction with a KRAB domain-containing proteins bound to DNA, followed by binding to HP1 proteins to facilitate heterochromatin formation. As with other TIF1 family proteins, TRIM28 functions as an E3 ubiquitin ligase, and in 2020, Wantanabee et al. developed a new E3 ligase substrate-trapping strategy in order to identify new ubiquitin substrates. They found that TRIM28 was associated with “Krüppel-associated box” proteins as expected, but also regulated cyclin A2 and transcription factor II beta (TFIIB) by mediating their degradation via ubiquitination [223]. Interestingly, the PHD finger and bromodomain region of TRIM28 do not appear to contribute to chromatin recognition. Instead, the PHD finger of TRIM28 was found to function as an E3 ligase that SUMOylates the adjacent bromodomain [224]. Furthermore, SUMOylation of the bromodomain is necessary for KRAB-mediated gene repression, and functions to recruit the SET Domain Bifurcated Histone Lysine Methyltransferase 1 (SETDB1) and the chromodomain helicase DNA-binding protein 3 (CHD3) subunit of the NuRD histone deacetylase complex to silence transcription [224]. Thus, TRIM28 activity contributes to the development of several cancers. For example, TRIM28 is overexpressed in glioma [225], cervical cancer [226], lung cancer [227], hepatocellular carcinoma [228], and breast cancer [50]. Overexpression of TRIM28 is often associated with a more aggressive disease and poor patient outcomes, but it is unclear if bromodomain inhibition would be an effective therapeutic strategy since it does not appear to function as a canonical chromatin reader domain.

TRIM33 was first identified as a potential regulator of embryonic development [229] and hematopoiesis [230]. More recently, TRIM33 was found to function as the main monoubiquitin ligase of Smad4 (Mothers Against Decapentaplegic homolog 4), which is essential for the cellular signaling activity of the transforming growth factor beta (TGFβ) [231]. This finding indicates TRIM33 functions to antagonize the Smad/TGFβ signaling pathways that control cellular developmental processes and homeostasis. The inactivation of TRIM33 has been found to contribute to the development of a variety of cancers. For example, the expression of TRIM33 is downregulated in Pancreatic Ductal AdenoCarcinoma (PDAC), and it was shown that the inactivation of TRIM33 contributes to the development of cystic pancreatic tumors through the activation of *Kras* [232]. TRIM33 activity has also been linked to breast cancer and leukemia progression [233,234]. The C-terminal PHD finger-Bromodomain module of TRIM33 has been shown to function as a multivalent chromatin reader. The PHD finger preferentially recognizes the unmodified histone H3, while the bromodomain binds to the histone H3 with di-acetylation modifications, particularly H3K18acK23ac [235]. In addition, methylation of histone H3 at the R2 or K4 positions inhibited the interaction of the TRIM33 PHD-Bromo with histone ligands [235]. Importantly, the recognition of acetylated histones activates the E3 ligase activity, and the PHD-Bromodomain region, in addition to the RING domain, is essential for TRIM33 to ubiquitinate Smad4 [235]. A more recent study also showed that the PHD-Bromo cassette forms a single functional unit that preferentially recognizes histone H3 that is tri-methylated at K9, and contains multiple downstream lysine acetylation modifications (e.g., H3K9me3K14acK18acK23ac) [236]. Since TRIM33 also recruits Smad2/3 recognition of PTM chromatin, it is thought to poise the TRIM33/Smad complexes at active response elements to stimulate cellular differentiation through TGFβ signaling pathways. These strong connections to cancer-promoting pathways suggest that the TRIM proteins would be excellent candidates for therapeutic targeting, and more research is needed to develop effective inhibitors for this protein class.

Lastly, the TRIM bromodomain proteins have well-characterized roles in the DNA damage response (DDR) [237,238,239] (also recently reviewed in McAvera et al., 2020 [240]). The DDR is carried out by a network of factors that sense DNA damage and rapidly signal the recruitment of chromatin remodeling and DNA repair machinery to sites of DNA damage. A prior study used laser micro-irradiation to create localized DNA damage in cells, and systematically evaluated the recruitment of 32 GFP-tagged bromodomains to sites of DNA damage using fluorescence microscopy [241]. They showed that a group of 12 bromodomain proteins including the TRIM bromodomains, TRIM24, TRIM28, and TRIM33, localize to sites of damage. Histone acetylation plays a central role in recruitment of the DDR machinery [242]. For example, it was observed that the levels of H3K9ac and H3K56ac decrease following DNA damage, and these levels are later restored after DNA repair [243]. The rapid reduction in H3K56 acetylation levels occurs through the recruitment of histone deacetylase enzymes (HDAC1/2) to sites of double-stranded DNA breaks [244]. Several studies have since shown that histone H4K56ac marks are an important signal at cell cycle check points, and function as a regulator of genomic stability [245,246]. A specific mechanism for the role of H3K56ac in DDR was recently elucidated in embryonic stem cells where TRIM66 was shown to recognize this modification [209]. The binding of TRIM66 to unmodified H3R2-H3K4 in combination with H3K56ac resulted in the subsequent recruitment of the histone deacetylase SIRT6 to lower the acetylation levels and initiate DDR [209]. Thus, this bromodomain-dependent mechanism facilitates the assembly of DNA repair proteins, and highlights how chromatin reader proteins can contribute to the maintenance of genomic integrity. The link between TRIM proteins and the DDR may also provide an opportunity to utilize combination therapies targeting dual pathways that contribute to cancer development.

### 4.8. Speckled Protein Family of Bromodomain Proteins

The speckled protein (SP) family of bromodomain proteins includes SP100, SP110, SP140, and the SP140-like protein (SP140L) [247]. The SP proteins have an N-terminal caspase activation and recruitment domain (CARD), which is typically involved in oligomerization, and is found in a wide variety of proteins that often contribute to apoptosis, the inflammatory response, or immunogenic signaling [248]. In SP proteins, the CARD domain is speculated to be involved in homodimerization, but its role is poorly characterized in terms of its function in immune cells [247]. The SP family proteins also contain several functional domains that implicate them as chromatin readers. The SAND domain (named according to proteins that have it: SP100, Aire, NucP41/P75, and DEAF) has been shown to bind to DNA and mediate protein–protein interactions [249,250]. The C-terminal PHD finger and bromodomain function in histone recognition.

Speckled 100 kDa (SP100) is a nuclear protein that was discovered in association with promyelocytic leukemia nuclear bodies (PML-NBs) in patients with primary biliary cirrhosis [247,251]. SP100 is alternatively spliced into 11 different isoforms, and it is also modified via several post-translational modifications including acetylation, phosphorylation, and ubiquitination [252,253]. These modifications likely contribute to regulating the cellular functions of SP100. PML bodies are membrane-less nuclear structures that are often associated with stressed cellular states, including viral infection, oxidative stress, and DNA-damage [254,255]. The PHD-Bromodomain region has been shown to recognize histone H3 that is unmethylated at K4 (H3K4me0), while the bromodomain plays a structural role in stabilizing the PHD fold, rather than recognizing acetylated lysine [256]. Although SP100 was shown to preferentially bind unmodified histone H3, its binding was permissive of other adjacent modifications including phosphorylation of Thr3 (H3T3ph), tri-methylation of K9 (H3K9me3), and phosphorylation of S10 (H3S10ph) [256]. These results indicate that histone recognition by the PHD-bromodomain, in addition to DNA binding by the SAND domain, may be an important mechanism for targeting the SP100 protein to specific chromatin regions to regulate transcription.

Similar to SP100, the SP140 and SP140L proteins are autoantigens in primary biliary cirrhosis, and SP140 is active in chronic lymphocytic leukemia [247]. The PHD-bromodomain cassette found in SP140 preferentially recognizes unmodified histone H3K4me0; however, its binding was disrupted in the presence of methylation at either H3K4 or H3K9 [256]. Interestingly, the SP140 protein appears to be regulated via SUMOylation, and the PHD-Bromodomain is a SUMOylation target of SUMO-1, with the PHD finger facilitating binding of the Ubc9 E2 ligase and SUMO-1 to stimulate SUMOylation of the adjacent bromodomain [257]. The SP family of proteins is involved in several aspects of the innate cellular immunity pathways [247]. Although inhibitors are not currently available for this class of bromodomain-containing proteins, there may be an opportunity to target them for immunosuppressive applications in the future.

### 4.9. Zinc Finger MYND Bromodomain Proteins

Two bromodomain-containing proteins within the family VII also possess a unique zinc finger—the MYND (Myeloid, Nevery, and DEAF1) motif. ZMYND8 (also known as RACK7) was first identified as a receptor of protein C kinase, and ZMYND11 (alternatively BS69) was originally recognized as a suppressor of human adenovirus E1A activated genes [258,259]. Both proteins share structural architecture consisting of an N-terminal PHD-BRD-PWWP arrangement followed by a C-terminal MYND domain [260,261].

ZMYND8 has since been shown to play a role in many cellular functions including the DNA damage response, [241] where it promotes homologous recombination and DNA repair. The PHD-BRD-PWWP triple reader domain is important for the recruitment of ZMYND8 to specific cellular locations, and for interactions with transcriptional complexes such as the NuRD complex, and the co-repressor of the RE1-silencing transcription factor complex (Co-REST) [262]. The PHD finger associates with histone H3 that is unmodified, and coordination of the N-terminus of histone H3 is particularly important for driving the binding interaction. Histone H3K14ac is a preferred ligand of the tri-valent PHD-BRD-PWWP module, as is the histone H3K36me2/3, and the histone H4K12ac modifications [260,262]. The bromodomain was found to be responsible for the coordination of histone H4, while the PWWP domain recognizes histone H3K36me3 as well as DNA [262]. Both histone and DNA interactions were necessary for the recruitment of ZMYND8 to sites of DNA damage [262]. Furthermore, the recognition of chromatin modifications is tied to cancer development. ZMYND8 typically functions as a tumor suppressor, and binding to the H3K4me1-H4K14ac modifications has been found to inhibit the expression of genes associated with cancer metastasis [49]. ZMYND8 activity has also been shown to be protective in triple negative breast cancer [263] and nasopharyngeal carcinoma [263]. In addition, the activation of ZMYND8 expression through the all-trans-retinoic acid (ATRA) has been shown to inhibit cancer cell proliferation [264]. ZMYND8 promotes genes associated with terminal differentiation in opposition to the maintenance of cancer stem cells [265]. This stemness characteristic of cancer cells is associated with the development of resistance to chemotherapies and relapse. Overexpression of ZMYND8 was shown to re-sensitize cells to chemotherapy via the recruitment of the EZH2 methyltransferase and lysine demethylase KDM5C corepressors to the promoters of tumor oncogenes. This resulted in an altered chromatin state, enriched in the repressive transcription mark H3K27me3 [265]. However, recent reports have indicated that ZMYND8 may also play an oncogenic role. For example, the hypoxia inducible factors 1 and 2 (HIF-1 and HIF-2) were induced by ZMYND8 in human breast tumors, and this was correlated with poor patient outcomes [54]. This oncogenic activity appears to be controlled via a switch between the gene repressor to gene activator functions of EZH2, that is, regulated by phosphorylation of EZH2 [266]. Thus, ZMYND8 may prove to be an attractive therapeutic target in a subset of cancer patients [267].

ZMYND11 appears to act as a negative regulator of transcription, similarly to ZMYND8. However, ZMYND11 is a significant contributor to cancer development. For example, copy number variations of ZMYND11 were found in clinical samples of patients with several types of hematological malignancies [268], and it is fused to the malignant brain tumor domain containing 1 (MBTD1) protein via a chromosomal translocation associated with acute myeloid leukemia [269,270]. ZMYND11 has been shown to specifically bind H3.3K36me3, and its histone-binding activity is sensitive to changes in amino acid substitutions in the histone tail as well as adjacent PTMs [271]. Histone H3.3 contains a serine at position 31, and the replacement of this residue with an alanine in histone H3.1 and H3.2, or phosphorylation of serine 31 in histone H3.3, significantly weakened the bind binding interaction [271]. This is due to specific binding contact formed by the dual Bromo-PWWP domain that creates a critical hydrogen bond contact with S31 in histone H3.3 [271]. Furthermore, knockdown of ZMYND11 resulted in upregulation of c-Myc, promoting transcription and cellular proliferation [271]. Interestingly, mutations in histone H3.3 are oncogenic and are associated with pediatric brain cancers [272]. The histone H3K36M and H3G34R/V mutations result in decreased binding of ZMYND11 to histone H3.3 due to a lack of tri-methylation at lysine 36. In addition, the loss of the H3K36 methyltransferase SETD2 in several cancers also results in impaired chromatin interactions by ZMYND11 [273]. These interactions highlight the crucial role of ZMYND11 as an important tumor suppressor and demonstrate how the interplay in epigenetic modifications contribute to cancer development.

## 5. Emerging Strategies to Target Bromodomain Proteins

The discovery of JQ1 and I-BET as potent and selective inhibitors for the BET family of bromodomains shifted the paradigm for the chromatin reader field. Prior to the development of JQ1, it was thought that bromodomain inhibitors would have broad activity towards all human bromodomain-containing proteins, resulting in extensive off-target effects. Instead, JQ1 was shown to selectively target the bromodomains of BRD4, BRD3, and BRD2 with nanomolar binding affinities, while also having very little activity towards non-BET bromodomains [27]. Furthermore, the use of JQ1 in a mouse model of nuclear protein in testis (NUT) midline carcinoma, which results from a chromosomal fusion of BRD4 with NUT, resulted in improved survival and tumor regression [27]. Similarly, I-BET was shown to preferentially bind to the bromodomain of BRD4, followed by BRD3, and BRD2, and disrupted their interaction with tetra-acetylated histone H4 ligands. These early studies stimulated a broad interest in understanding the structure and functions of bromodomains, and current advances include the characterization of the structures and histone ligand binding activities for nearly all of the human bromodomains [31]. Several investigations into the mechanism of action for BET bromodomain inhibitors have provided new insights into the protein interaction networks of these proteins with both histone and non-histone proteins [45]. For example, BRD3 was shown to be a binding partner of the GATA1 transcription factor via recognition of specific acetyllysine modifications, and the addition of a BRD inhibitor disrupted this interaction [47]. Importantly, BET bromodomain inhibition was identified as a therapeutic strategy in multiple myeloma, working to inhibit the transcription of the c-Myc oncoprotein, which resulted in cellular senescence and halted proliferation of leukemia cells [274]. BET inhibition has also been used to study the role of these bromodomains in HIV infection, leukemogenesis, and spermatogenesis [275,276,277]. Numerous BET inhibitors have been evaluated, or are currently undergoing evaluation, in clinical trials. Importantly, while JQ1 is an essential tool compound for studying BET bromodomain function(s), it has been extensively modified for use in the clinic. JQ1 derivatives, such as OTX015 (Birabriseb), have demonstrated greater therapeutic efficacy. Successful targeting of the BET bromodomain family spurred a global interest in the development of additional bromodomain inhibitors targeting both BET and non-BET bromodomain proteins. While the inhibition of BET bromodomains for cancer therapy has remained a highly active area of research, there are now chemical probes available to specifically target individual bromodomain-containing proteins from all eight bromodomain subfamilies [278]. Table 1 provides a current summary of promising BETi and non-BET inhibitors that show potential in the clinical setting.

Understanding the biological relevance, structural uniqueness, and clinical applications of BRD inhibition are important steps in the development of BRD inhibitors as cancer therapeutics. Although the downregulation of MYC transcription has been widely proposed as a key mechanism for BET inhibitor anti-tumor activity [274], additional mechanisms for BET inhibitor efficacy against cancer have also been reported. For example, it has been demonstrated in models of B-cell lymphoma that BET inhibition modulates the expression of pro- and anti-apoptotic BCL-2 family members to induce apoptosis through intrinsic mitochondrial apoptotic pathways [305,306]. The efficacy of BET inhibitors as anti-cancer agents has been evaluated in preclinical studies for multiple cancer types, with drugs presently at different stages of clinical trials (Table 1). However, as with most targeted cancer therapies, resistance to inhibitors limits their effectiveness in patients. A variety of mechanisms underlying resistance to BET inhibitors have been reported for different tumor types. BET inhibitor resistance has been widely reported to occur through the reactivation of MYC expression. For example, in a study on the development BET inhibitor resistance in acute myeloid leukemia, Fong C et al. discovered that stimulation of the Wnt/beta-catenin pathway resulted in increased binding of beta-catenin at *MYC* regulatory sites where BRD4 was displaced from the chromatin. This appears to prime a subset of the leukemia stem cells for transcriptional plasticity allowing them to upregulate *MYC* and take over as the dominant cell type harboring BET inhibitor resistance [204]. In triple-negative breast cancer, resistance to JQ1 treatment emerged through altered epigenetic signaling of BRD4. In the resistant cells, higher levels of phosphorylated BRD4 were detected, and this led to increased binding interactions of BRD4 with MED1, resulting in decreased responsiveness to bromodomain inhibition [280]. Another study found that the voltage-dependent anion channel 1 (VDAC1) is also linked to the development of resistance to JQ1 in breast cancer [307]. More recently, a comprehensive study by Shu et al. carried out a genome-wide CRISPR screen to identify genes that contribute to the development of resistance after JQ1 treatment in triple-negative breast cancer cell lines. Importantly, they discovered additional therapeutic agents that are synergistic with JQ1 in inhibiting tumor cell growth. These included DNA-damaging agents (doxorubicin) and microtubule inhibitors (Paclitaxel/Vincristine). They found that Palbociclib had the most significant effect and worked by enhancing CDK4 inhibition-mediated G1 arrest and by destabilizing BRD2/4 via proteasomal degradation [308]. Several other studies have also found the cellular effects of bromodomain inhibition in cancer treatment to be enhanced by combination therapy. Due to their ability to intrinsic apoptosis signaling, recent reports demonstrate synergistic activity of BET inhibitors and the small-molecule BCL-2 inhibitor ABT199/venetoclax in killing *MYC*-driven B-cell lymphoma cells [306,309,310]. For example, the combined inhibition of both BET bromodomains and HDAC enzymes improved the efficacy of either drug class alone [311,312]. This led to the prediction that using bromodomain inhibitors in addition to the standard therapy may reduce the development of resistance in some cancers [313]. Combinations of epigenetic therapeutics have also proven successful in the treatment of acute myeloid leukemia [314], multiple myeloma [315], pancreatic cancer [316], ovarian cancer [317], and in breast cancer [318]. One mechanism for this synergy has been outlined through the dual inhibition of BET bromodomains in combination with poly[adenosine diphosphate (ADP)-ribose] polymerase inhibitors (PARPi). The addition of a bromodomain inhibitor blocks the homologous recombination (HR) DNA repair pathway in addition to the base excision repair pathway, sensitizing HR proficient cancers [319].

Another therapeutic strategy that has shown great promise to improve BRD inhibitor efficacy is the targeted degradation of bromodomain-containing proteins. Proteolysis-targeting chimeras (PROTACs) were first developed in 2001 by Sakamoto et al., in order to direct disease causing proteins for ubiquitin-dependent degradation by the proteosome [320]. The key feature of the chimeric molecule is the linker, which contains a BRD ligand mimic/small molecule on one end, and an E3 ligase recognition domain on the other. Thus, thalidomide derivatives will bring the bromodomain-containing protein of interest to the Cereblon E3 ligase, while a short peptide sequence from Hypoxia-inducible factor 1 (HIF1) will recruit the VHL E3 ligase. This has been done successfully with BRD4, BRD9, TRIM24, and PCAF/GCN5 [288,296,321,322]. A BRD9 specific degrader was developed as a tool compound to study the function of this bromodomain-containing protein [322], and it was demonstrated that degradation of BRD9 triggered downregulation of oncogenic programs that contribute to the development of synovial sarcoma/soft tissue tumors [81]. A similar approach was used with TRIM24, where the protein is targeted for selective degradation by chemically conjugating the small-molecule inhibitor IACS-9571 to the Von Hippel–Lindau (VHL) E3 ubiquitin ligase [296]. Degradation of the entire TRIM24 protein was shown to have a more immediate and longer-lasting impact on cellular proliferation than BRD inhibition alone, particularly in leukemia cell lines [296].

The advantage of using PROTACs to degrade bromodomain-containing proteins is that these proteins are often targeted to the chromatin via multiple domains. The result is that some BRD inhibitors are not as effective as expected [104]. For example, BRD inhibitors developed for SMARCA2/4 did not produce the expected anti-proliferative effects [127,133]. Thus, degraders to disrupt the formation of active SMARCA2/4 ATPase complexes were designed as an alternative treatment strategy. This resulted in significantly reduced protein levels and increased apoptosis, suggesting that targeted degradation of these complexes may be an attractive therapeutic strategy [293]. Furthermore, PROTACs have proven to act like a catalyst, working many times in a row to degrade multiple proteins. Their effect is rapid even at low concentrations, and the duration of activity is sustained over time since the cell must re-synthesize the protein of interest [323]. PROTACS taking advantage of BET inhibitors such as JQ1 and OTX015 (Birabriseb) have been evaluated in various cellular models of cancer including prostate cancer, lymphoma, and leukemia [287,288,324]. Importantly, since bromodomain inhibitors have been shown to increase the stability of BRD4, degradation has proven to be an effective method to overcome resistance [308].

## 6. Conclusions

Currently, cancer therapy includes a mixture of surgical, radiation, and drug therapies [325]. Recent epigenomic profiling studies using various breast cancer cell models have revealed the distinctive super enhancer landscapes of breast cancer subtypes [326,327]. Cell type–specific enhancers can become deregulated to allow the downstream activation of genes that promote tumorigenesis and metastasis [328]. Functional genomic and transcriptomic profiling has revealed the differential expression patterns of bromodomain genes across many cancer types. Overall, bromodomain proteins are largely dysregulated in their expression across tumor versus normal tissues for most cancers. Specific bromodomain proteins are now correlated with aggressive cancers and can be used as biomarkers for cancer progression. Bromodomain-containing proteins remain an exciting drug target, and structure and functional studies on BET and non-BET proteins have made significant advances in moving this field forward. Over the past decade, the development of selective bromodomain inhibitors for use alone, in combination therapy, or as a catalyst for targeted protein degradation, have provided additional insights into bromodomain protein function in normal cellular processes and in disease progression. Furthermore, analysis of the protein–protein interaction networks of bromodomain-containing proteins has highlighted the cellular signaling pathways they are involved in, which provides novel insights on how multiple pathways can be targeted in cancer. The highly diverse activities of BRD proteins, and the distinct ways in which they interact with chromatin and other proteins to regulate cellular activities, emphasize the need for highly selective inhibitors targeting the BRD binding pocket, and adjacent functional domains, to act as effective therapeutic agents. Indeed, while BRDi’s have continued to work as tool molecules for understanding the roles of specific BRD proteins, as these compounds have moved into the clinic, the development of resistance to BRDis has added to the complexity of their use as therapeutic agents. Current advances in combinatorial inhibitor treatments and chimeric PROTACs have improved the efficacy of BRDi’s in clinical settings. It can be expected that more combinatorial therapeutic options will be developed to circumvent the development of drug resistance in cancer, with BRD inhibitors emerging as key players in combinatorial therapies, targeting the underlying epigenetic regulatory networks across various cancer types.

## Figures and Tables

**Table 1:** Summary of bromodomain (BRD) inhibitors with potential in the clinical setting.

BRD Inhibitor (Molecule Images Created with JSME [279])		Target	Cancer Type/Results	Clinical Trial ID/Reference
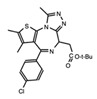	JQ1	BET’s	The first generation of BET inhibitors which has proven to be a valuable tool for understanding BET BRDs in numerous cancers, but demonstrated toxicities in the clinic. As a result derivatives of JQ1 have had greater clinical success.	[27,280,281]
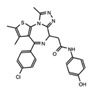	OTX015* (Birabresib)	BET’s	Identifiers and resulting publications for ongoing and completed clinical trials for OTX015 as an exclusive therapy or in combination treatment. This JQ1 derivative used in combination with PROTACs has shown promise in cell models of prostate cancer, lymphoma, and leukemia.	NCT02698176, NCT02259114, NCT01713582, NCT02698189, NCT02296476 [282,283,284,285,286,287,288]
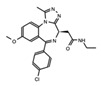	I-BET762 GSK525762A, (Molibresib)	BET’s	Phase 1 clinical trial of this orally available compound initially showed that daily dosing with molibresib was well tolerated and showed efficacy for patients with nuclear protein in testis (NUT) carcinoma.	[28,288,289,290]
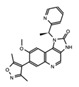	I-BET151 GSK1210151A	BET’s	This BET inhibitor demonstrates strong anti-proliferative effects, and xenograft models indicate repression of proliferation in myeloma cells. However, this drug has not made progressed to clinical trials.	[291]
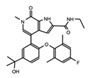	ABBV-744	Pan-BET (Selective for the 2nd bromodo-main)	Selective for the 2nd bromodomain of the BET-bromodomain proteins, and has demonstrated anti-proliferative effects for numerous acute myeloid leukemia and prostate cancer cell lines.	NCT04454658 [110,111]
Not available	ZEN-3694	BET’s	A current phase 2 clinical trial using ZEN-3694 in combination with the enzalutamide is recruiting for castration resistant prostate cancer (CRPC), where the compound has shown efficacy in a phase 1 clinical trial.	NCT04471974 [292]
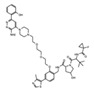	ACBI1	SMARCA2/4	This PROTAC degrader resulted in reduced protein levels and apoptosis of acute myeloid leukemia (AML) cells.	[293]
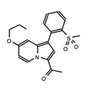	GSK2801	BAZ2A/B	The selective acetyl-lysine competitive inhibitor induces apoptosis in triple negative breast cancer (TNBC) cells in combination with BET inhibitors.	[144,294]
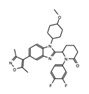	CCS1477	CBP/p300	Current Phase 1 & 2 clinical trials are recruiting patients for treatment of hematological malignancies and advanced prostate cancer.	NCT03568656, NCT04068597
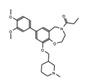	I-CBP112	CBP/p300	Combination therapy with the p300/CBP active site inhibitor (A-485) resulted in reduced p300 chromatin enrichment, and decreased expression of androgen-dependent and pro-oncogenic genes in leukemia and prostate cancer.	[154,295]
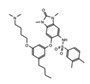	IACS-9571	BRPF1/TRIM24	This selective inhibitor has provided insights into cellular functions, and may be useful as a potential therapeutic for acute myeloid leukemia (AML) and breast cancer (BCa).	[165]
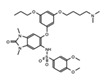	IACS-9571	TRIM24	When developed into a bifunctional degrader linked to the VHL E3 ligase, TRIM24 protein degradation resulted in a greater negative impact on proliferation in leukemia cell lines.	[296]
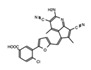	AM879	ATAD2	Treatment with AM879, prevented cell proliferation, and induced apoptosis in triple negative breast cancer (TNBC) cells.	[195]
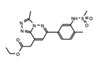	Bromospo-rine	Multi-BRD (BET)	Shows promise as a therapeutic for colorectal cancer (CRC) when administered in combination with 5-Fluorouracil (5-FU).	[12,297]
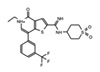	I-BRD9	BRD9	The selective inhibitor identified cancer associated and immune response genes as possible targets of BRD9 regulation in leukemia cells.	[298]
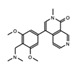 BI-7273 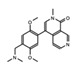 BI-9564 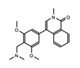 BI-7271 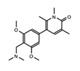 BI-7189	BI-7273/BI-9564 BI-7271/BI-7273/BI-7189	BRD9	These small molecule inhibitors display anti-tumor activity in xenograft models of AML.	[299]
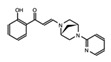	PFI-3	SMARCA2/4 and PB1(5)	Treatment with PFI-3 has been shown to sensitize cancer cells to chemotherapeutic agents.	http://www.thesgc.org/chemical-probes/PFI-3, accessed on 7 July 2021 [300]
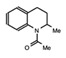 MS2126 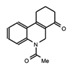 MS7972	MS2126/MS7972	CBP/p300	Cell based assays in osteosarcoma cells, demonstrated that these molecules can modulate the p53 response to DNA damage.	[301]
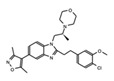	SGC-CBP30	CBP/p300	Inhibition of CBP is suggested to be a potential method for targeting transcriptional dependencies in multiple myeloma.	[302,303,304]
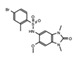 OF-1 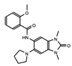 PFI-4 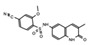 NI-57	OF-1, PFI-4, NI-57	pan-BRPF	While these inhibitors have not be linked to anti-proliferative or anti-cancer therapies, they have been suggested as potential therapeutics in bone malignancies.	[167]

## Supplementary Materials

The following are available online at https://www.mdpi.com/article/10.3390/cancers13143606/s1: Table S1: Bromodomain protein–protein interactions list. This table contains all the interactions associated with BRD-containing proteins. For each interaction in the table, at least one of the nodes (proteins) is a bromodomain-containing protein. This table can be loaded directly to Cytoscape or RStudio to retrieve the interactions of specific proteins.
